# SYSTEMATIC LITERATURE REVIEW ON MODELS AND PRACTICES OF EARLY CHILDHOOD INTERVENTION IN BRAZIL

**DOI:** 10.1590/1984-0462/;2017;35;4;00015

**Published:** 2017-09-21

**Authors:** Bruna Pereira Ricci Marini, Mariane Cristina Lourenço, Patrícia Carla de Souza Della Barba

**Affiliations:** a Universidade Federal de São Carlos, São Carlos, SP, Brazil.

**Keywords:** Early intervention, Childhood, Rehabilitation, Development, Health, Intervenção precoce, Infância, Reabilitação, Desenvolvimento, Saúde

## Abstract

**Objective:**

To identify which Early Childhood Intervention practices and models are described in the Brazilian literature.

**Data sources:**

A systematic integrative review of the literature indexed in databases from Virtual Health Library, Bielefeld Academic Search Engine, Education Resources Information Center and Portal of Periodicals of the Coordination of Improvement of Higher Education Personnel was carried out, considering the period between 2005 and 2015. The following articles were analyzed: those published in English or Portuguese, fully available online, with the terms “Early Intervention”, “Early Stimulation” or “Essential Stimulation” in the title, abstract or keywords; studies that enrolled children aged from 0 to 6 years, their caregivers or professionals in Early Intervention services; manuscripts published in journals classified as ≥ B2 (WebQualis; Qualis 2014) in the fields of Education or Physical Education; and studies that described Early Intervention practices.

**Data synthesis:**

Early Intervention seems to be developed exclusively related to the health sector, with prevalence of practices aimed at stimulating skills through the use of clinical approaches, whose focus is centered on the child and structured in the model of rehabilitative care.

**Conclusions:**

The adoption of Early Intervention practices and models are far from those recommended and recognized by the international literature as good practices. In this sense, the need of continuous education of professionals involved in this area is shown, as well the need for investments in research on this subject.

## RESUMO

**Objetivo:**

Identificar quais práticas e modelos de Intervenção Precoce na Infância estão descritos na literatura científica brasileira.

**Fontes de dados:**

Realizou-se uma revisão sistemática integrativa da literatura indexada entre 2005 e 2015 nas bases Biblioteca Virtual em Saúde, *Bielefeld Academic Search Engine, Education Resources Information Center* e Portal de Periódicos da Coordenação de Aperfeiçoamento de Pessoal de Ensino Superior. Foram incluídos artigos publicados em língua inglesa ou portuguesa, na íntegra e disponibilizados *online* que: contivessem os termos “Intervenção Precoce” ou “Estimulação Precoce” ou “Estimulação Essencial” no título, resumo ou palavras-chave; contassem como participantes crianças de 0 a 6 anos, seus cuidadores ou profissionais de serviços de Intervenção Precoce; estivessem indexados em uma revista com classificação igual ou superior a B2, segundo avaliação do WebQualis (Qualis 2014), para as áreas de Educação ou Educação Física; e descrevessem práticas de Intervenção Precoce.

**Síntese dos dados:**

Verifica-se que a Intervenção Precoce parece desenvolver-se exclusivamente aliada ao setor da saúde, com prevalência de práticas voltadas à estimulação de habilidades, com enfoque centrado na criança, estruturadas a partir de um modelo reabilitativo de cuidado e do emprego de abordagens clínicas.

**Conclusões:**

Os resultados apontam para a adoção de práticas e modelos de Intervenção Precoce distantes daqueles recomendados e reconhecidos como boas práticas pela literatura internacional. Nesse sentido, aponta-se a necessidade da atualização dos profissionais que atuam nessa área, bem como do investimento em pesquisas que incidam sobre a temática.

## INTRODUCTION

The long years of study about human development led to the consensus that it is constituted as a lifelong process of growth and physical, psychic and social maturity, which, influenced by the cultural and historical contexts to which the subjects are exposed, results in a great variety of individual differences.^1^ Therefore, development can be seen as a dynamic, continuous and progressive process through which the individual acquires and perfects skills related to several contexts.^2^

Even though these constructions and acquisitions happen continuously throughout life, the first childhood is pointed out as a crucial period for development, due to the fast structural and brain maturation, to the higher neural plasticity and to the development of essential skills that will be the base for more complex gains.^2^ ^,^ ^3^ ^,^ ^4^ ^,^ ^5^ However, if the acquisitions of that phase are determinant, so are the intercurrences.

During the first childhood, children can be exposed to a series of factors that will have a negative impact on their development, known as risk factors. These factors can be constituted by direct threats, such as the exposure to infectious agents and injuries, among others; or by the lack of opportunities, generated by social inequality, poverty and racism.^6^ In this sense, it is observed that some deficits that take during childhood may turn into more complex problems with time, if not solved immediately, leading to more chances of lacking personal, political, economic and social resources toward their resolution. This shows the need for an intervention that is able to work on these conditions as soon as possible.^2^

In this context, Early Intervention (EI) practices are pointed out as beneficial for children exposed to risk factors, and are also recommended for children with developmental disorders and impairment.^4^ ^,^ ^7^ ^,^ ^8^ Guralnick^7^ ^,^ ^8^ highlights that the EI practices are considered as an important resource by professionals from different countries.

 From the beginning of the processes to structure EI programs, a series of theoretical and conceptual transformations was incorporated as a result of the advancements in the knowledge about childhood development, resulting in a range of services with different characteristics.^4^ ^,^ ^5^ ^,^ ^9^ ^,^ ^10^ At first, these services were based on medical model practices, addressed to the diagnosis and treatment of the difficulties found, using specific protocols and an agenda focused on the children, emphasizing their “socialization outside the family context, the search for better understanding childhood development and practical applications of developmental theories”, according to Shonkoff and Meisels.^10^

After the 1970s, with the expansion of EI programs and studies that proved their efficacy, there were transformations in the models of care, so the attention turned also to the family. In the 1980s, such transformations were strengthened by the contributions of the Ecological Models of Human Development and the Transactional Model of Development, resulting in a new approach of systemic, ecological EI, focused on the family, favoring actions conducted within a transdisciplinary work perspective.^4^ ^,^ ^11^

In this context, the focus on the family stands out as one of the main approaches of the theoretical and conceptual evolutions that took place in the XX century, leading to a new scenario of practices in which families began to be included as partners of care promotion professionals, with the understanding of development as a result of broader processes.^4^ ^,^ ^10^ According to Simeonsson and Bailey,^12^ the evolution of this process can be analyzed in phases, whose features concern the level of parental participation and the professional conduct in this relationship, leading to different focuses attributed to practice.

Based on these concepts, EI can be understood as actions of specialized support addressed to children and families who, throughout the first childhood, present with difficulties regarding development and social inclusion. From this perspective, its objectives are based on the effectiveness of the leading role of the family by strengthening its competences in terms of child care, and in the provision of services and resources that promote their social inclusion and development.^13^

Guralnick^14^ mentions that ten practical principles are recommended to ensure good practices in EI programs:

1. A structure of development that involves all components in an EI system;
2. Integration and coordination of all EI services;
3. Inclusion and participation of children and families in community activities and programs;
4. Early detection and identification of risk factors;
5. Surveillance and control of development as part of the system;
6. Planning of individualized interventions for each case;
7. Evaluation of services and interventions;
8. Development of culturally appropriate interventions;
9. Adoption of practices based on evidence;
10. Maintenance of systemic perspective.

However, despite the advancements in knowledge and the evidence and prestige obtained by the EI model focused on the family, studies show differences between the recommended practices and those executed in the services, as well as different ways to structure care. It varies according to context, conducted practices and theoretical reference models; this scenario points to the need for studies addressed to the problem of EI practices.^15^ ^,^ ^16^

In Brazil, even though the appearance of EI programs began in the 1970s, it seems like the subject is little discussed, and this matter reflects even on the use of different terms as synonyms to refer to this type of service. In this sense, Bolsanello^17^ indicates that the shortage of national research and scientific productions about EI may have a direct impact on the practices carried out, leading to a service that apparently does not correspond to that recommended internationally.

Therefore, based on the need to conduct further studies to clarify the scenario of EI in Brazil, this question guided this study: What EI practices and models are described in the national scientific literature?

## METHOD

Considering the objectives of this study, we used the methodology of integrative and systematic literature review, which consists of gathering and synthetizing, systematically, the scientific knowledge that has already been produced about a specific subject, enabling the broad understanding of the analyzed problem.^18^ Therefore, this study was elaborated according to the six phases recommended for the elaboration of a high-quality integrative review:^18^ ^,^ ^19^ ^,^ ^20^ ^,^ ^21^

1. Identification of the theme and selection of the research question;
2. Establishment of inclusion and exclusion criteria;
3. Identification of pre-selected and selected studies;
4. Categorization of the selected studies;
5. Analysis and interpretation of results;
6. Presentation of the review/synthesis of knowledge.

The phase of identification of pre-selected and selected studies was conducted by two independent researchers, in order to guarantee scientific rigor. The following databases were used to select the articles in the sample: Virtual Health Library (BIREME); Bielefeld Academic Search Engine (BASE); Education Resources Information Center (ERIC) and the Portal of Periodicals of the Coordination of Improvement of Higher Education Personnel (CAPES).

The selection of descriptors to be used was made considering the variety of terms used as synonyms in the Brazilian context. Therefore, the following descriptors were used (in Portuguese): “Early intervention”, “Early Stimulation”, and “Essential Stimulation”, in a simple association with the term “Childhood Development”, as well as the terms in English Early Intervention, Child Development and Brazil.

The inclusion criteria adopted were: papers published in English or in Portuguese, full version, available online; papers published from 2005 to 2015; including the terms “Early Intervention”, “Early Stimulation” or “Essential Stimulation” in the title, in the abstract or in the keywords; whose participants were children aged from 0 to 6 years, their caretakers of EI service professionals; to be indexed in a journal classified as B2 or higher, according to the evaluation of WebQualis (Qualis 2014) for the fields of Education or Physical Education; and describing EI practices.

For the phase of selection and categorization of studies, we elaborated a listing matrix in which we organized the data referring to each study. For the analysis and interpretation of results, texts were read in full, and a summarized matrix was elaborated for the qualitative evaluation of the information, containing: complete reference, objective of the study, intervention studied, approach of the intervention and model.

The results and discussion are presented descriptively, using the exposure of data regarding the publications and the analysis of their content.

## RESULTS AND DISCUSSION

**Figure 1:**
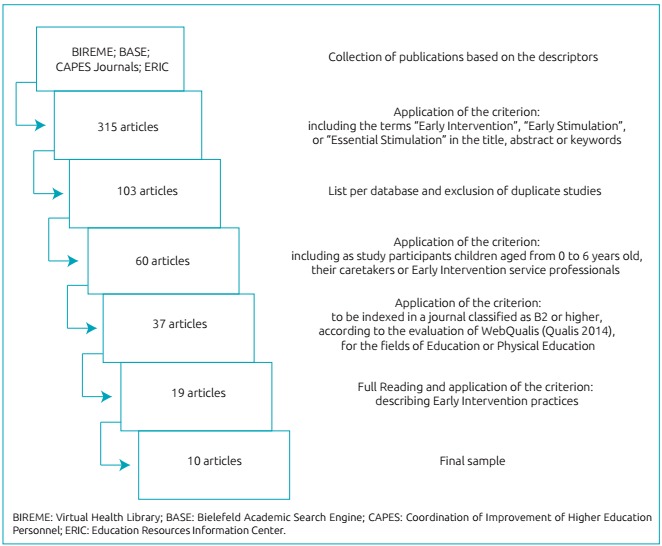
Flowchart of the stage of study selection.

The identification of the pre-selected publications for this study began with the collection of publications in the described databases; after using the descriptors, 315 papers were chosen. Based on that, we selected those studies that corresponded to the criterion: having the terms “Early Intervention”, “Early Stimulation”, or “Essential Stimulation” in the title, abstract or keywords - and 103 articles were selected. These articles were listed separately in a sheet, according to the database and the descriptors used for recovery. After the listing, data were crossed and the ones in duplicity were excluded, resulting in 60 papers. These were analyzed according to the criterion: including participants aged from 0 to 6 years old, their caretakers or EI service professionals - and then, 37 studies were selected. Afterwards, this criterion was used: to be indexed in a journal classified as B2 or higher, according to the WebQualis evaluation (Qualis 2014), for the fields of Education or Physical Education - resulting in 19 papers, who were read in full. Finally, based on the analysis of the entire content, we selected the ones which met the criterion of describing EI practices, and that resulted in 10 papers that composed the final sample (Figure 1). ^22^ ^,^ ^23^ ^,^ ^24^ ^,^ ^25^ ^,^ ^26^ ^,^ ^27^ ^,^ ^28^ ^,^ ^29^ ^,^ ^30^ ^,^ ^31^

Using the data generated in the listing matrix, it was observed that, among the ten papers: three were indexed in BIREME; three, in BASE; two were simultaneously in BIREME and BASE; and two were simultaneously in BASE and in CAPES. Based on the search terms, no papers were taken from the ERIC base. As to the year of publication, we selected: 1 article (10%) from 2008, 2 (20%) from 2009, 3 (30%) from 2012, 2 (30%) from 2013 and 1 (10%) from 2014. In this search, we did not identify papers published in 2005, 2006, 2007, 2011 and 2015. These results corroborate with papers that show the lack of national publications about the theme.^17^ Another important factor shown by the results refers to the annual distribution of publications, which reveals the instability in the analysis of this subject, since we observed long periods without any publication in the studied bases.

The scientific journals in which the studies were published are: *Revista Brasileira de Crescimento e Desenvolvimento Humano*, with 1 article (10%); *Distúrbios da Comunicação*, with 2 articles (20%); *Motricidade* (Santa Maria da Feira), with 1 article (10%); *Movimento* (from Universidade Federal do Rio Grande do Sul - UFRGS), with 1 article (10%); *Educar em Revista*, with 2 articles (20%); *Revista de Terapia Ocupacional da USP*, with 1 article (10%); *Estudos de Psicologia*, with 1 article (10%); and *Psicologia em Estudo*, with 1 article (10%). Regarding the professional experience of the authors, we identified 3 occupational therapists (12%), 6 psychologists (24%); 4 professionals of Physical Education (16%); 4 speech-language pathologists (16%); and 8 physical therapists (32%).

Among the studies, 4 (40%) used a quantitative approach; 5 (50%) used a qualitative approach; and 1 (10%) used a mixed approach. It was also possible to identify that 8 studies (80%) applied the observational design, and 2 (20%), the experimental design. In this sense, Cândido et al.^32^ hypothetize that the small number of studies, which, in fact, implement a proposal of intervention, is owed to the difficulties related to this type of work: “Papers of this kind are conducted, but not in an investigative manner, which may somehow influence the advances in the knowledge in the fields of Early Intervention”

Five studies (50%) included participants who were exclusively children aged between 0 and 6 years, and, in 3 of them, the children presented with special needs (Down syndrome, cerebral palsy, congenital blindness, neuropsychomotor developmental delay). Two studies (20%) included children and their parents, and, in this case, they all had special needs. In 2 of them (20%), participants were the parents or caretakers of children with special needs; and, in 1 study (10%), there were professionals who carried out EI actions.

The interventions reported were carried out in different scenarios: daycare facility (3 studies), philanthropic institution (1 study), university hospital (2 studies), household of the participants (2 studies), public maternity wards (1 study), and center of studies and research related to the university (1 study).

**Chart 1:**
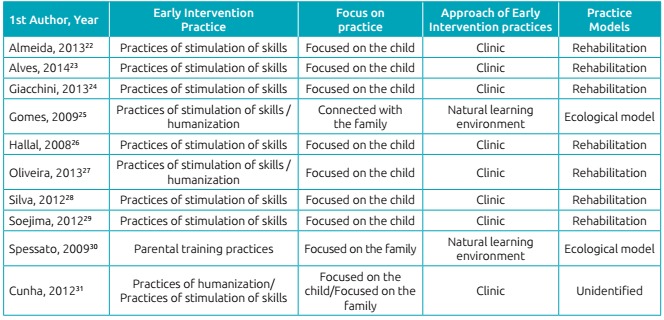
Categorization of data referring to the publications.

Based on the analysis of the data comprised in the summarized matrix, the studies were classified in categories, containing subcategories and units of analysis, as follows:

1. EI Practices:
  1. Practices of stimulation of skills;
  2. Parental training;
  3. Practices of humanization;
2. EI practice approach:
  1. Clínic;
  2. Based on participation;
3. Focus of the practice:
  1. Focused on the child;
  2. Connected with the family;
  3. Focused on the family;
4. Practice Models:
  1. Rehabilitation;
  2. Ecological (Chart 1).

### Early Intervention Practices

**Chart 2:**
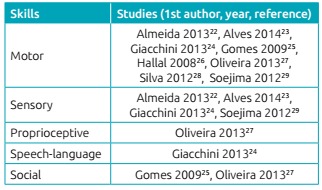
Distribution of stimulation practices according to the skills focused in the studies.

Regarding EI practices, we identified the prevalence of practices of stimulation of skills, described in 9 out of the 10 analyzed studies. This subcategory included those of motor, sensory, proprioceptive, speech-language and social stimulation (Chart 2), prescribed by professionals based on the characteristics and needs identified in the children. The observation was mostly made based on developmental scales or standardized instruments that assess specific areas in which intervention was predicted. Parental training was identified as an EI practice in two studies; however, it was used with different goals: one was addressed to train parents for stimulation in the household^24^, and the other focused on training to change patterns of didactic interactions with their children during playtime.^30^ Practices of mother-child bond were also described as an EI practice. In the analysis by Cunha and Benevides,^31^ the authors identified the EI practices conducted by psychologists in maternity wards, according to the understanding these professionals have on the subject. In their results, the reception, the maternal listening and the perception of the baby as a subject are described as EI practices, once they impact on possible risk factors related with child development. Humanization practices are also described in the study by Gomes and Duarte^25^ as EI. These authors implement the intervention focusing on the transformation of the hospital environment, by including ludic activities to provide “opportunities of motor and social stimulation”, as well as the resignification of the hospital space.

### Approach of Early Intervention practices

It is observed that the practices discussed are mostly constituted of clinical approaches (described in eight of the tem studies), based on the identification and intervention regarding conditions of developmental deviation, focusing on the child’s impairments. It is worth to mention that such structure is similar to that described by Bolsanello,^17^ referring to a mechanistic work behavior, limited to the stimulation of deficit and not considering the children in their broader aspect. Therefore, according to the reports, for 40 years professionals have been limited to describing the practices regarding the child, exclusively, without including the family and the community in the process. However, it is important to mention that an effort has been identified in terms of changing these approaches, as shown in the studies by Silva and Aiello^28^ and Gomes and Duarte,^25^ who adopt the approach based on participation. The referred approach is based on the incorporation of services to the routines and daily activities of the family, and on the promotion of learning through opportunities of participation of the children and by teaching efficient strategies for parents and caretakers to interact in a positive manner with the children,^33^ ^,^ ^34^ therefore valuing the abilities of their family members and their own as important instruments for intervention. This type of approach is in accordance with the good practices proposed in EI, spread globally, according to which the interventions should focus on the family, with the objective of strengthening the family function in order to identify and promote their competences, not only thinking of rehabilitation.^9^ ^,^ ^35^

### Focus on Early Intervention practices

As a reflex of the approaches described, it is observed that practices focusing on the children were mostly used, and were identified in nine of the analyzed studies. According to Serrano,^4^ this focus begins with a paradigm instituted in the early XX century, according to which “in the center of the difficulties of the children was their personality or genetic disorders inherited from the parents”. Therefore, the assessment and intervention are focused exclusively on the child, addressing special attention to his or her biological and psychic characteristics and to their impact on deelopment.^4^ ^,^ ^34^ This focus is knowingly applied on services that adopt a “traditional” model of EI, in which addressed interventions are used in order to generate learning opportunities and practice of skills. This model has been pointed out as being directly opposite to the recommended practices.^34^

Despite the prevalence of the focus centered on the child, we identified studies, as well as in the analysis of the approaches, that incorporate proposals that are close to those described as good EI practices, using broader focuses - that is, attention is also addressed to the family. In this sense, in the scope of the studies analyzed, there was one focusing on “connected with the family”^24^, and two “focusing on the Family”.^31^ ^,^ ^25^ The practices classified as “connected with the family” concern those in which the parents are instruments for the professionals, such as co-therapists, implementing the interventions prescribed or “trained”, whereas the ones “focusing on the family” consider them to be consumer of services, providing them with options of intervention so that they can choose whichever fits their problem best.^36^ In the studies mentioned, there are advances in the process of inserting the family in care, starting with the recognition of the child as a part of a system, and considering the influence of this system on his or her development. However, it is not yet possible to state the existence of practices focusing on the family, once the professionals are still in the center of care, as holders of the knowledge and responsible for the interventions, and the needs of the children are still guiding these practices. Therefore, it is possible to observe that the focus of care remains addressed to the needs of the children, even in cases when the family is involved. In this sense, the literature indicates that high-quality EI, as currently conceived, should focus on the families and on the needs identified by it, functioning as a facilitator in the process of strengthening family competences, as well as the network of formal and informal support aiming at promoting family autonomy, towards the satisfactory resolution of its needs. ^9^ ^,^ ^37^

### Practice Models of Early Intervention

Based on the data regarding the practices listed in this study, the rehabilitation model was more common, identified in seven of the tem studies, against only two analyses that used the ecological model. It is important to mention that, in one of the studies, it was not possible to identify the practice models, once they were not described in detail.

Regarding the models used, even though only five studies were based on the ecological perspective, quoting the work of Brofenbrenner as an introduction reference, the development of these studies points to the difficulty to incorporate interventions that are actually ecological. Therefore, it is important to think about how many aspects should be analyzed in order to guarantee the implementation of ecological actions, taking the risk to reproduce the main object of criticism of Brofenbrenner:^38^ “the science of the unknown behavior, of the child in unknown situations, with unknown adults, for as brief periods of time as possible”.

## CONCLUSIONS

Based on the analysis of the national production about the subject, it is verified that practices and EI models seem to develop exclusively when allied with the health sector, with strong prevalence of practices addressed to the stimulation of skills, using clinical approaches structured from a rehabilitating care model, focusing on the child.

These characteristics, associated with the lack of literature about the theme and the existing conceptual divergence, point to the need for a national effort regarding the professional update and the adoption of practices that are similar to those recommended and recognized as good practices by the international literature. There is also the need for higher investments in studies about the theme, starting with the recognition of its importance and the existing scientific gap. It is worth to mention that, in this sense, there have been efforts, however, it seems urgent to potentialize and spread them.

Therefore, this study is expected to collaborate with the discussions related with EI practices in Brazil, based on its contribution to elucidate the scenario in the past ten years. Here, we point to the need for further studies about the EI practices, as well as about the conceptual gaps, in order to cooperate and form a theoretical group about EI in Brazil.

## References

[1] Papalia DE, Olds SW, Feldman RD (2006). Desenvolvimento Humano.

[2] Comitê Científico do Núcleo Ciência pela Infância (2014). O Impacto do Desenvolvimento na Primeira Infância Sobre a
Aprendizagem.

[3] Guralnick MJ, McCartney K, Phillips D (2006). Family influences on early development: Integrating the science of
normative development, risk and disability, and intervention. Blackwell handbook of early childhood Development.

[4] Serrano AM (2007). Redes Sociais de Apoio e a sua Relevância para a Intervenção Precoce.

[5] Fernandes MD (2001). Subsídios para a caracterização de programas de intervenção precoce
implementados pelas equipas de apoios educativos na região de
Trás-Os-Montes.

[6] Garbarino J, Ganzel B, Shonkoff JP, Meisels SJ (2000). The Human Ecology of Early Risk. Handbook of Early Childhood Intervention.

[7] Guralnick MJ (2015). Merging Policy Initiatives and Developmental Perspectives in Early
Intervention. Escr Psicol.

[8] Guralnick MJ (2016). Early Intervention for Children with Intellectual Disabilities: An
Update. J Appl Res Intellect Disabil.

[9] Pimentel JV (2005). Intervenção Focada na Família: desejo ou realidade.

[10] Shonkoff JP, Meisels SJ (2000). Handbook of Early Childhood Intervention.

[11] Camacho MJ (2010). Editorial. Diversidades.

[12] Simeonsson RJ, Bailey BB, Meisels SJ, Shonkoff JP (1990). Family dimensions in early intervention. Handbook of Early Childhood Intervention.

[13] European Agency for Development in Special Needs Education (2010). Early Childhood Intervention: progress and developments 2005-2010.

[14] Guralnick MJ (2008). International perspectives on early intervention: a search for common
ground. J Early Interv.

[15] Bairrão J, Almeida IC (2003). Questões actuais em Intervenção Precoce. Psicologia.

[16] Graça PR, Teixeira ML, Lopes SC, Serrano AM, Campos AR (2010). The moment of assessment in early intervention: study of parental
involvement of psychometric properties of ASQ-2 from 30 to 60
months. Rev Bras Educ Espec.

[17] Bolsanello MA (2003). Concepts about intervention and assessement strategies in early
intervention professionals. Educ Rev.

[18] Whittemore R, Knafl K (2005). The integrative review: Updated methodology. J Adv Nurs.

[19] Sampaio RF, Mancini MC (2007). Systematic review studies: a guide for careful synthesis of the
scientific evidence. Rev Bras Fisioter.

[20] Grupo Anima Educação (2014). Manual Revisão Bibliográfica Sistemática Integrativa: a pesquisa baseada em
evidências.

[21] Gomes IS, Caminha IO (2014). Guia para estudos de revisão sistemática: uma opção metodológica para
as ciências do movimento humano. Movimento.

[22] Almeida CS, Valentini NC (2013). Nurseries environment and the intervention in babies'
development. Motricidade.

[23] Alves PV, Sousa GA, Gagliardo HG (2014). Functional abilities in children with congenital blindness: a case
study. Rev Ter Ocup Univ São Paulo.

[24] Giacchini V, Tonial A, Mota HB (2013). Aspects of language and oral motor observed in children treated at an
early stimulation sector. Distúrb Comun.

[25] Gomes CA, Duarte E (2009). Mother-infant play: essential stimulation for children with cerebral
palsy. Estud Psicol.

[26] Hallal CZ, Marques NR, Braccialli LM (2008). Acquisition of functional abilities in the mobility area by children
assisted in an early stimulation program. Rev Bras Crescimento Desenvolv Hum.

[27] Oliveira LD, Peruzzolo DL, Souza AP (2013). Early intervention in a case of prematurity and risk for development:
contributions of the proposal of a single therapist, supported in
Interdisciplinarity. Distúrb Comun.

[28] Silva NC, Aiello AL (2012). Teaching the father how to play with his baby with Down's
syndrome. Educar em Revista.

[29] Soejima CS, Bolsanello MA (2012). Early intervention program in nursery school with
babies. Educ Rev.

[30] Spessato BC, Valentini NC, Krebs RJ, Berleze A (2009). Early childhood education and motor intervention: a view based on the
bioecological theory of Bronfenbrenner. Movimento.

[31] Cunha AC, Benevides J (2012). Psychologist practice in early intervention in the maternal and child
health. Psicol Estud.

[32] Cândido AR, Cia F (2014). Análise da produção nacional de estudos sobre identificação e intervenção
precoce.

[33] Campbell PH (2004). Participation-Based Sevices: promoting children´s participation in
natural settings. Young Exceptional Children.

[34] Campbell PH, Sawyer LB (2007). Supporting learning opportunities in natural settings through
participation-based services. J. Early Interv.

[35] Gronita J, Matos C, Pimentel JS, Bernardo AC, Marques JD (2011). Intervenção Precoce: o processo de construção de boas práticas.

[36] Dunst CJ, Johanson C, Trivette CM, Hamby D (1991). Family-oriented early intervention policies and practices:
family-centered or not?. Except Child.

[37] Dunst CJ, Bruder MB (2002). Valued Outcomes of Service Coordination, Early Intervention and
Natural Environments. Except Child.

[38] Bronfenbrenner U (1996). A Ecologia do Desenvolvimento Humano: Experimentos Naturais e
Planejados.

